# A community-based study of dental fluorosis in rural children (6–12 years) from an aspirational district in Karnataka, India

**DOI:** 10.3389/fpubh.2023.1110777

**Published:** 2023-03-16

**Authors:** U. Venkateswara Prasad, Phaniraj Vastrad, Chandan N., Manish J. Barvaliya, Rahul Kirte, Sabarinath R., Suman K. Ray, Ravichandran B., Tapas Chakma, Manoj V. Murhekar, Subarna Roy

**Affiliations:** ^1^Model Rural Health Research Unit, Department of Health Research (Government of India), Sirwar, Raichur, Karnataka, India; ^2^National Institute of Traditional Medicine, Indian Council of Medical Research (ICMR), Belagavi, Karnataka, India; ^3^Raichur Institute of Medical Sciences, Raichur, Karnataka, India; ^4^National Institute of Epidemiology, Indian Council of Medical Research (ICMR), Chennai, Tamil Nadu, India; ^5^Regional Occupational Health Center, Indian Council of Medical Research (ICMR), Bangalore, Karnataka, India; ^6^National Institute of Research in Tribal Health, Indian Council of Medical Research (ICMR), Jabalpur, India

**Keywords:** dental fluorosis, fluoride level, drinking water, oral health, school-going children

## Abstract

**Objectives:**

The present study was planned to estimate the prevalence of dental fluorosis in 6–12 years of children and its association with various drinking water sources, water, and urine fluoride levels among the subset of children under the umbrella of a larger study to address iodine deficiency disorders and iron deficiency anemia in 17 villages of Manvi and Devadurga talukas of Raichur district of Karnataka.

**Methods:**

Analysis of subset of data and urine samples of children under the umbrella of a larger cross-sectional community-based study was conducted in 17 villages of Manvi and Devadurga taluks of Raichur district. House to house survey was carried out to collect data using a semi-structured questionnaire in ODK software. Demographic details, source of drinking water, clinical assessment of dental fluorosis, and height and weight measurements were performed by trained staff. Urine and water samples were collected for fluoride level estimation. The overall prevalence of dental fluorosis and its severity-wise prevalence were estimated. Association between dental fluorosis and age, gender, type of diet, source of drinking water, height for age, BMI for age, water fluoride level, and urine fluoride level were carried out using logistic regression analysis.

**Results:**

The prevalence of dental fluorosis was 46.0%. Mild, moderate, and severe dental fluorosis was found in 37.9, 7.8, and 0.3% of children. With the increasing age of participants, the odds of dental fluorosis were found to increase by 2–4 folds. The odds of having dental fluorosis were significantly increased with increasing water fluoride levels of 3 to 5 ppm [AOR = 3.147 (1.585–6.248); *P* = 0.001] in comparison with water fluoride levels of < 1 ppm. The similar trend was found with urine fluoride level > 4 ppm [AOR = 3.607 (1.861–6.990); *P* < 0.001]. As compared to river water, other sources of drinking water were significantly associated with higher odds of dental fluorosis.

**Conclusions:**

Prevalence of dental fluorosis was high in 6 to 12 years due to overexposure of fluoride from drinking water. High water and urine fluoride levels in children indicate the chronic exposure to fluoride and suggest that the population is at high risk of developing chronic fluorosis.

## Introduction

Dental fluorosis is a disorder of dental enamel due to repeated exposure to high fluoride concentrations during tooth development, resulting in enamel with lower mineral content and more porosity and it is common in pediatric individuals (1, 2). Fluorosis ranges from inconspicuous white spots or streaks to heavy brown stains in middle of the teeth away from the gum margin and pitted enamel. Fluoride is considered to be double edged weapon and overexposure to fluoride is always dangerous. Fluorosis is prevalent in many regions of the world and is brought on mainly by excessive fluoride in drinking water (3). The consumed fluoride remains in the human body for a long time, although about 80% of it is eliminated in urine (4, 5). Biomarkers of fluoride help to detect insufficient or excessive ingestion of fluoride. Urine fluoride concentration among the biomarkers of fluoride exposure is generally regarded as the best indicator as it can be collected non-invasively and also, the concentration of fluoride in urine reflects the burden of fluoride exposure from drinking water (6).

According to WHO recommendations from 1984, the ideal level of fluoride (F^−^) in drinking water should be kept below 1.5 ppm (1.5 mg/L) in tropical climates (7). Similarly, according to the Bureau of Indian Standards (BIS), 1 ppm (1.0 mg/L) of fluoride is the maximum desired level in drinking water (8), however lesser the better.

Dental fluorosis is a problem in 15 states of India with high fluoride levels in drinking water (9). During the 11th five-year plan, the Government of India started the “National Programme for Prevention and Control of Fluorosis” (NPPCF) as a new health effort to deal with the fluorosis problem in the country (10). Karnataka is one of the fluoride-endemic states in India, and several of its districts are said to have significant levels of fluoride in their groundwater (11). According to NPPCF data, areas with a high prevalence of dental fluorosis include Mysore, Bellary, Chikkaballapur, Koppal, Davangere, Tumkur, Bagalkot, Bangalore (U), Bijapur, Raichur, Chitradurga, Gadag, Gulbarga, Hassan, Kolar, Mandya, Ramanagaram, and Shimoga (10). A high prevalence of dental fluorosis is associated with high fluoride concentration in drinking water and urine. A higher level of urine fluoride is associated with high exposure to fluoride, and it may increase the severity of dental fluorosis in exposed persons (12). Because the damage and changes to the bones and teeth caused by long-term exposure to high fluoride levels are permanent, fluorosis care puts a lot of focus on prevention, and health promotion.

Raichur is located in the northeastern region of Karnataka State of India. In this area, groundwater is a significant source of drinking water. The prevalence of dental fluorosis in six villages of Raichur taluk and district was reported at 32.6% in school children (5 to 10 years) a decade ago in 2011 (13). The data on dental fluorosis and its association with sources of drinking water, and fluoride levels in water and urine are scarce in Karnataka, especially in Raichur. Identifying the magnitude of problem and for further guidance of taking remedial measures, this study was planned to estimate the prevalence of dental fluorosis in 6–12 years of children and to find out its association with various drinking water sources, water, and urine fluoride levels amongst the subset of children from a larger study of addressing the issues of iodine deficiency disorders and iron deficiency anemia in 17 villages of Manvi and Devadurga talukas of Raichur district of Karnataka.

## Methodology

### Study design and setting

**Figure 1:**
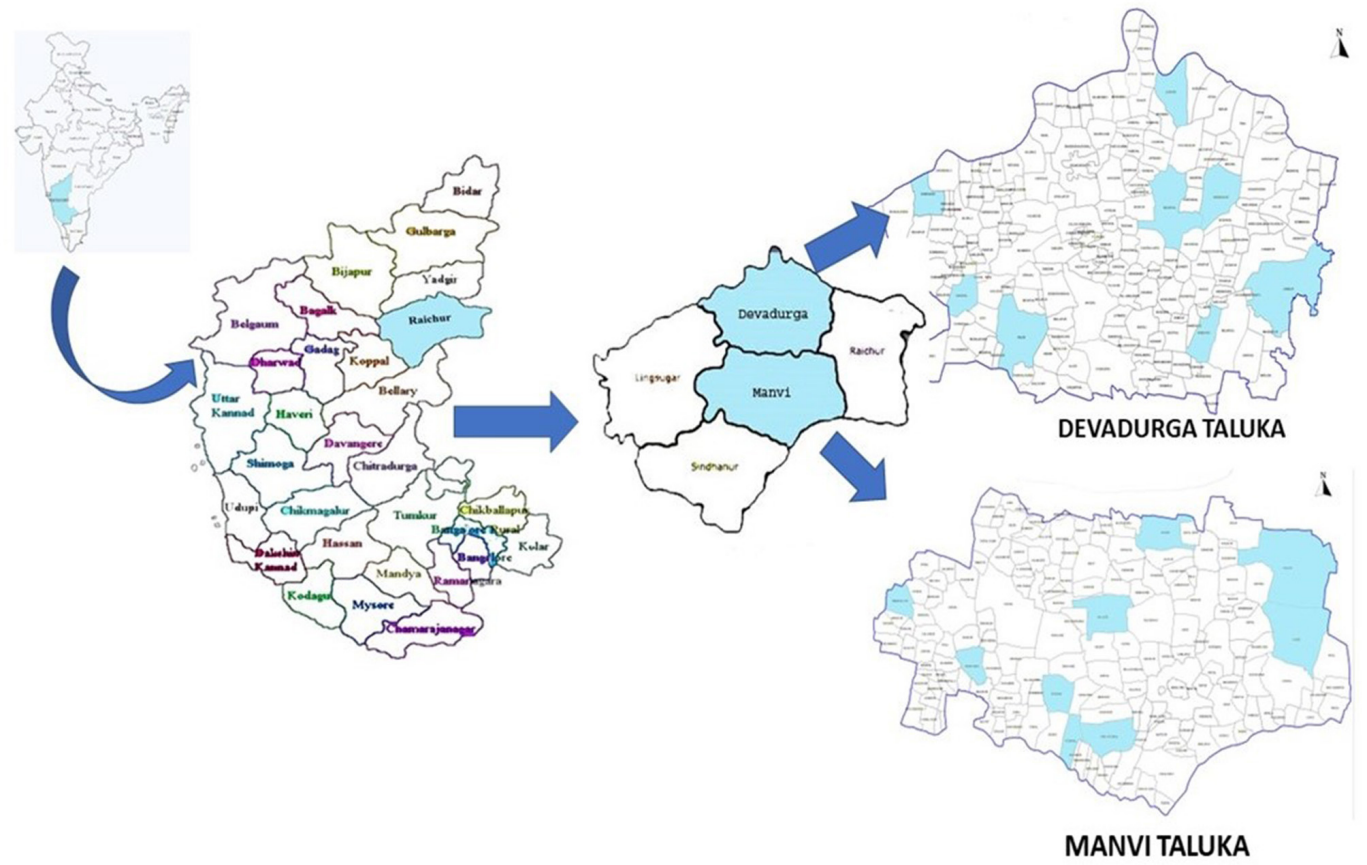
Geographice details of the study area.

This study was conducted under the umbrella of a larger study to address iodine deficiency disorders and iron deficiency anemia at the Model Rural Health Research Unit (MRHRU), Raichur. The present community-based cross-sectional study was carried out in two of the most backward taluks of the aspirational district (Raichur) i.e., Manvi and Devadurga (Figure 1). There are a total of 17 Primary health centers (PHCs) in these two taluks: 9 in Manvi and 8 in Devadurga. The village under each PHC location was included in the present study. Children aged 6 to 12 years residing in these villages whose parents consented to participate were included after taking their verbal assent. Children in whom dental fluorosis assessment was not possible due to extrinsic stains on the teeth were excluded.

### Sample size

The clinical examination of fluorosis was carried out for all the children (*n* = 1,614) whereas, urine fluoride estimation was performed in 649 eligible (≥ 30 ml) samples after performing urine iodine estimation in 1,614 urine samples.

### Data collection and sampling method

House to house survey was conducted and the households were visited by trained project staff along with Anganwadi/ ASHA workers of specific villages to conduct the study. The household was included in the study if it had children in the age group of 6–12 years and parents consented for their child to participate. In the case of a closed household, a household without a targeted study group and parents not consenting, the next household was contacted for participation in the study. If there were more than one child in the 6–12 years age group, the younger child was involved in the study and others were not. The data was collected in 2 months period (June and July 2021).

Before starting the data collection, the project staff was trained for conducting the interview, height and weight measurement, clinical examination for dental fluorosis and its grading, sample collection process, and sample transportation by the experts. Interviews were conducted with the parent/guardian, and data on the age, gender, diet, and source of drinking water were collected. Height and weight were measured by project staff using a SECA^®^ 213 portable stadiometer and SECA^®^ 813 digital flat scale.

**Figure 2:**
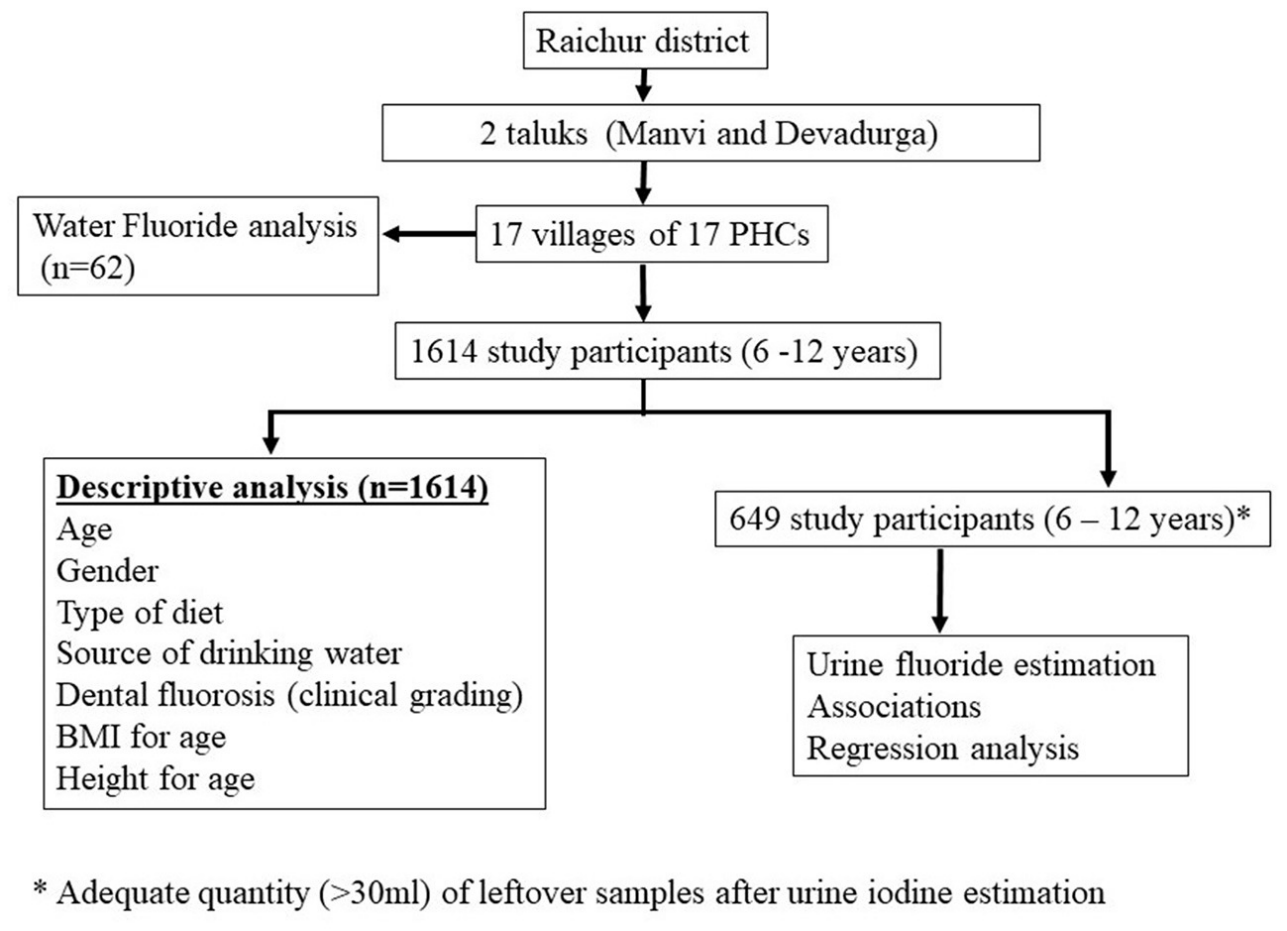
Flow of the study.

Children were asked to rinse their mouths with water before the examination of teeth for dental fluorosis. Clinical examination for dental fluorosis was done by trained project staff, and dental fluorosis was categorized into four categories according to the classification described by Haimanot et al. (14). The teeth were considered “normal” if there was no mottling and they were with a glazed white porcelain-like surface; teeth with white chalky opacities or patches on enamel with or without faint yellow lines were considered under the “mild fluorosis” category; distinct brown coloring of teeth was categorized as “moderate fluorosis”, and the presence of pitting or chipping of teeth was considered as “severe fluorosis”. The study flow has been depicted in Figure 2.

During the initial survey period, the supervisor randomly cross-checked 10% of the examinations done by project staff to ensure the correct categorization of dental fluorosis. The spot urine sample was collected in a non-reactive plastic sterile container with two drops of toluene, and the container was tightly closed by the project staff. The sample was labeled and put in an ice box and later transported to the MRHRU, Sirwar. The samples were stored in a refrigerator under 2 to 8°C. After urine iodine estimation, the samples with ≥ 30 ml volumes were subjected to fluoride estimation. The samples were coded and the person who decided the eligibility of urine samples for fluoride estimation was unaware of clinical data on fluorosis. On completing the required interviews in a particular village, the main community water sources were identified from the analyzed responses, and 30 ml water samples were collected in a non-reactive plastic sterile container from all listed sources. The water samples were labeled and transported to MRHRU, Sirwar. Both urine and water samples were analyzed for fluoride levels. From MRHRU, urine and water samples were transported in a cold chain to ICMR-National Institute of Research in Tribal Health (NIRTH) Jabalpur, and ICMR-Regional Occupational Health Center (ROHC) Bengaluru, respectively, for fluoride analysis.

### Fluoride level estimation

Fluoride ions in urine and water samples were measured using the procedures mentioned in the Orion instrument manual methods (15, 16). The analysis method is based on the Ion selective method using the ORION fluoride electrode (Thermo Scientific Orion 5-star Benchtop multi-parameter). Before running the test samples, a calibration curve was obtained from lower to higher concentrations (i.e., 0.1 ppm, 1 ppm, and 10 ppm) using the standard solutions for standardization. After standardization of the ORION Ion selective electrode and Ion meter, collected urine and water samples were run for fluoride analysis. Each urine sample was added with a 9:1 ml ratio of TISAB III (Total Ionic Strength Adjustment Buffer III, Thermo Scientific, India) into a 50 ml Plastic beaker and mixed well. After immersing the electrode into the prepared mixed solution and stabilization, the fluoride concentration was measured by an ORION ion meter. The electrode was washed with distilled water and wiped with dry tissue paper in each sample to avoid cross-contamination. In a similar way, each water sample was added with TISAB buffer II used for water fluoride analysis. We followed the same procedure for the 649 urine and 62 water samples and noted down the results of fluoride levels. Internal quality control was carried out for every 10th sample run. The measurement precision was assessed with a known addition method in urine samples and the mean fluoride recovery was calculated.

Water fluoride levels were categorized as < 1 ppm, 1 to 3 ppm, 3 to 5 ppm, and >5 ppm (NPPCF 2014). Urine fluoride levels were categorized as < 1 ppm, 1–2 ppm, 2–3 ppm, 3–4 ppm, and >4 ppm (12).

### Ethical consideration

Study documents were submitted for review and approval was taken from the Institutional Ethics Committee, Raichur Institute of Medical Science, Raichur for main large study and also, for subset analysis conducted in this manuscript (RIMS/IEC/Approval/Date 26-07-2021 and RIMS/IEC/Expedited Approval/ Date 23-11-2022 for No. 5/7/1656/CH/Adhoc/2019-RBMCH). Informed consent was obtained from the parents of the children and verbal assent was taken from study participants. The permission to conduct the study was obtained from District and State Administration.

### Statistical analysis

Data was collected using Open Data Kit (ODK) software using tablets and exported into a spreadsheet, and then analyzed using IBM Statistical Package for the Social Sciences (SPSS) V25. The prevalence of dental fluorosis was evaluated for 1,614 children whereas, the statistical analysis for an association was performed for 649 children. Descriptive statistics like frequency, percentages, mean, and standard deviation were used to describe the demographic data, water, and urine fluoride levels, prevalence of dental fluorosis, and grading of dental fluorosis. Height, weight, and BMI were converted into WHO Z scores by using WHO anthro plus software. The Chi-square test was used to find an association between age, gender, source of drinking water, type of diet, height for age, BMI for age, and urine fluoride levels with dental fluorosis grading. Bivariate and multivariate logistic regression was used to find an association between dental fluorosis with associated factors. The level of significance was kept at 0.05.

## Results

**Figure 3:**
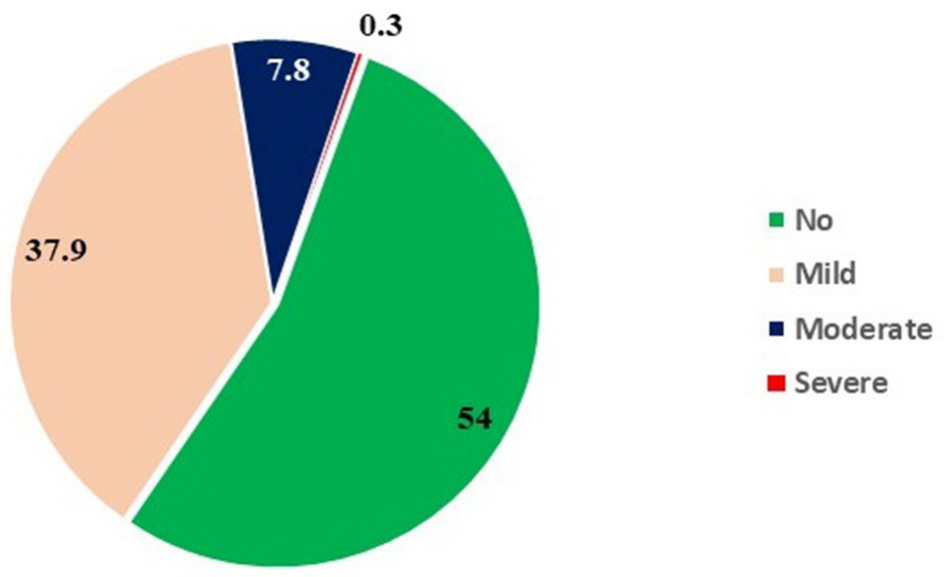
Proportions of various gradings of dental fluorosis in study participants (*n* = 1,614).

**Figure 4:**
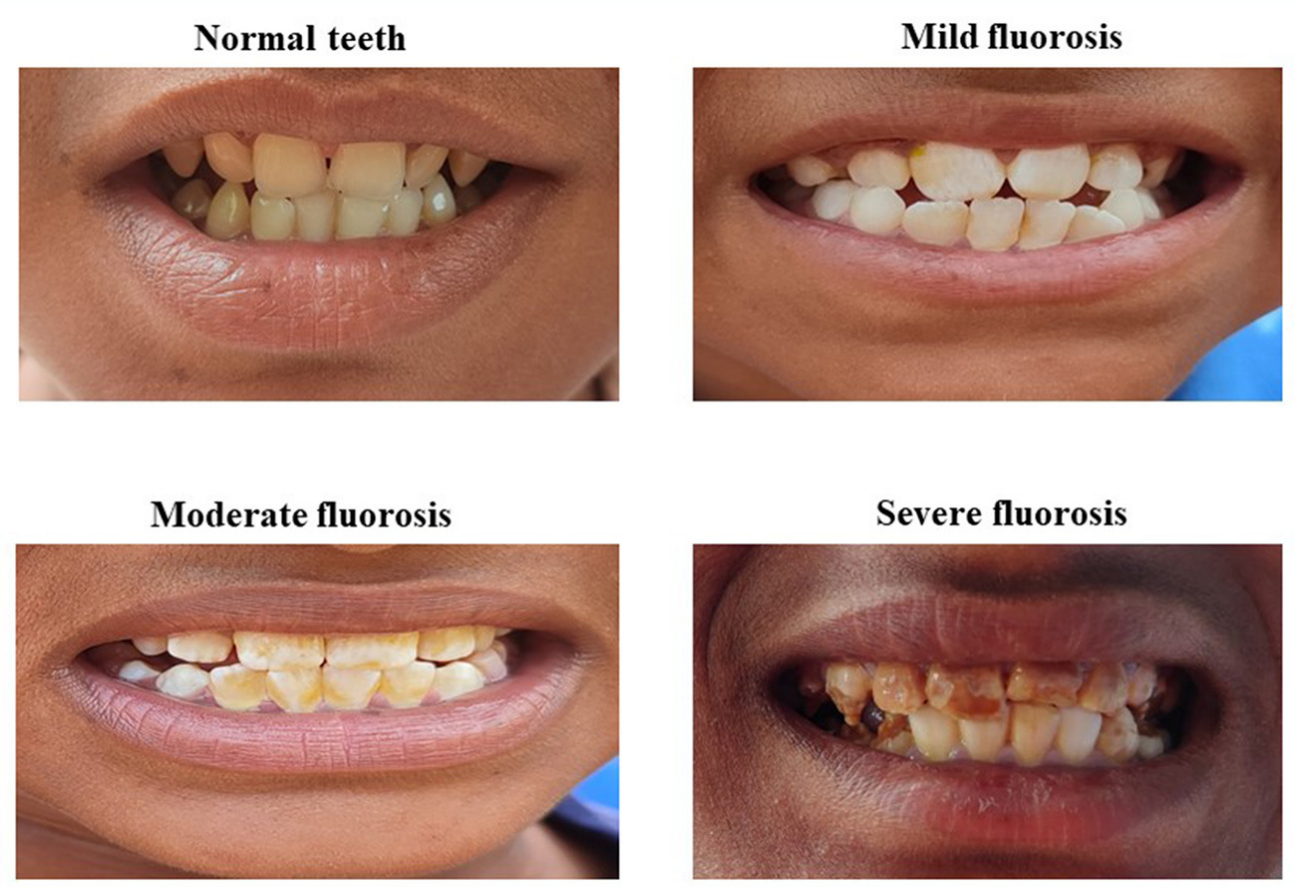
Images of participants with normal teeth and clinical grading of dental fluorosis.

The clinical evaluation was performed for 1,614 participants; while 649 urine samples and 62 water samples were analyzed. Amongst 1,614 children, 17.7, 14.4, 13.9, 14.1, 15.0, 11.5, and 13.4% were aged 6, 7, 8, 9, 10, 11, and 12 years, respectively. Females (53.3%) were more in comparison with males (46.7%). The prevalence of dental fluorosis was 46% in the study population (Table 1). The majority of cases of dental fluorosis were categorized into mild fluorosis (37.9%), with moderate 7.8% and severe fluorosis as 0.3% (Figure 3). Images of study participants with different clinical grading have been shown in Figure 4. Village-wise prevalence of dental fluorosis is shown in Table 2.

**Table 1:** Characteristics of study participants.

**Characteristic**	**Frequency**	**Percentage**
**Age (years) (*****n** =* **1,614)**		
6	286	17.7
7	233	14.4
8	225	13.9
9	227	14.1
10	242	15.0
11	185	11.5
12	216	13.4
**Gender (*****n** =* **1,614)**		
Male	753	46.7
Female	861	53.3
**Type of diet (*****n** =* **1,614)**		
Vegetarian	292	18.1
Mixed	1,322	81.9
**Source of drinking water (*****n** =* **1,614)**		
Borewell	90	5.6
River/ Pond	109	6.8
Tap water^*^	1,033	64.0
RO water	382	23.6
**Dental fluorosis on clinical assessment (*****n** =* **1,614)**		
Present	743	46.0
Absent	871	54.0
**Height for age (WHO Z scores)** ^**^(***n** =* **1,609)**		
Severely stunted (< -3SD)	148	9.2
Stunted (−3SD to −2SD)	332	20.6
Normal (−2SD to +2SD)	1,108	68.9
Tall (+2SD to +3SD)	14	0.9
Extremely tall (>+3SD)	07	0.4
**BMI for age (WHO Z scores)** ^**^(***n** =* **1609)**		
Severe thinness (< -3SD)	212	13.2
Thinness (−3SD to −2SD)	395	24.5
Normal (−2SD to +1SD)	957	59.5
Overweight (+1SD to +2SD)	31	1.9
Obese (+2SD to +3SD)	09	0.6
Extremely obese (>+3SD)	05	0.3
**Urine fluoride levels (*****n** =* **649)**		
< 1 PPM	121	18.6
1–2 PPM	224	34.7
2–3 PPM	139	21.4
3–4 PPM	70	10.8
>4 PPM	95	14.6

^*^May be from bore well/river/pond supplied from gram panchayat or municipality; ^**^Five participants' height and BMI were missing.

**Table 2:** Distribution of dental fluorosis in villages (*n* = 1,614).

**Dental fluorosis/ Villages**	**No [*n* (%)]**	**Mild [*n* (%)]**	**Moderate [*n* (%)]**	**Severe [*n* (%)]**
Torandinni (*n =* 88)	32 (36.4)	56 (63.6)	0	0
Ballatagi (*n =* 103)	85 (82.5)	17 (16.5)	01 (1.0)	0
Kallur (*n =* 117)	101 (86.3)	16 (13.7)	0	0
Pamankallur (*n =* 121)	52 (43.0)	67 (55.3)	02 (1.7)	0
Sirwar (*n =* 102)	48 (47.1)	49 (48.0)	05 (4.9)	0
Kurdi (*n =* 91)	74 (81.3)	15 (16.5)	02 (2.2)	0
Byagwat (*n =* 94)	76 (80.9)	18 (19.1)	0	0
Hirekotnekal (*n =* 100)	82 (82.0)	18 (18.0)	0	0
Pothnal (*n =* 71)	24 (33.8)	45 (63.4)	02 (2.6)	0
Gandhal (*n =* 91)	43 (47.3)	37 (40.7)	11 (12.0)	0
Gabbur (*n =* 97)	55 (56.7)	39 (40.2)	02 (2.1)	01 (1.0)
Galag (*n =* 106)	24 (22.6)	49 (46.2)	32 (30.2)	01 (0.9)
Koppar (*n =* 53)	22 (41.5)	20 (37.7)	11 (20.8)	0
Masarkal (*n =* 97)	51 (52.6)	38 (39.2)	07 (7.2)	01 (1.0)
Ramadurga (*n =* 80)	10 (12.5)	41 (51.2)	29 (36.3)	0
Hire budur (*n =* 99)	44 (44.4)	47 (47.5)	08 (8.1)	0
Chinchodi (*n =* 104)	49 (47.1)	39 (37.5)	14 (13.5)	02 (1.9)
Total (*n =* 1614)	872 (54.0)	611 (37.9)	126 (7.8)	05 (0.3)

Amongst 649 children whose urine samples were subjected to fluoride estimation, the majority were 6 (16.8%), 7 (16.2%), and 10 years (16.0%) old with a male-female ratio of 0.98. Of these, 546 (84.1%) children's diet type was predominantly non-vegetarian. Tap water (66.9%) was the most common source of drinking water, followed by community filters (21.7%), river or pond water (6.3%), and bore well (5.1%). Dental fluorosis based on clinical examination was present in 372 (57.3%) children out of 649 children. Urine fluoride levels of < 1, 1–2, 2–3, 3–4, and > 4 PPM were present in 121 (18.6%), 224 (34.7%), 139 (21.4%), 70 (10.8%), and 95 (14.6%) study participants, respectively. 441 (68.4%) children were having normal height, whereas 140 (21.6%), 56 (8.6%), and 08 (1.2%) children were stunted, severely stunted, and tall, respectively. On BMI for age WHO Z score assessment, 387 (59.6%) children had normal BMI; 159 (24.5%), 86 (13.3%), 07 (1.1%), and 06 (0.9%) children were thin, severely thin, overweight, and obese, respectively (Table 3). As shown in Table 3, age (χ^2^ = 40.678, df = 18, *P* = 0.002), source of drinking water (χ^2^ = 30.170, df = 9, *p* < 0.001) and urine fluoride levels (χ^2^= 37.181, df = 12, *p* < 0.001) were statistically significantly associated with dental fluorosis and its severity.

**Table 3:** Association between dental fluorosis with various factors.

**Variables**	**Dental fluorosis**				
	**Absent [*****n*** **(%)]**	**Mild [*****n*** **(%)]**	**Moderate [*****n*** **(%)]**	**Severe [*****n*** **(%)]**	**Measure of association**
**Age (*****n** =* **649)**					
6 (*n =* 109)	85 (78.0)	21 (19.3)	03 (2.7)	0	χ^2^= 40.678; df = 18; *P =* 0.002
7 (*n =* 105)	68 (64.8)	32 (30.5)	05 (4.7)	0	
8 (*n =* 87)	49 (56.3)	29 (33.3)	08 (9.3)	01 (1.1)	
9 (*n =* 87)	44 (50.6)	36 (41.4)	07 (8.0)	0	
10 (*n =* 104)	49 (47.1)	48 (46.2)	07 (6.7)	0	
11 (*n =* 70)	34 (48.6)	32 (45.7)	04 (5.7)	0	
12 (*n =* 87)	43 (49.5)	37 (42.5)	07 (8.0)	0	
**Gender (*****n** =* **649)**					
Male (*n =* 328)	179 (54.6)	128 (39.0)	20 (6.1)	01 (0.3)	χ^2^= 3.353; df = 3; *P =* 0.340
Female (*n =* 321)	193 (60.2)	107 (33.3)	21 (6.5)	0	
**Type of diet (*****n** =* **649)**					
Vegetarian (*n =* 103)	56 (54.4)	39 (37.8)	08 (7.8)	0	χ^2^= 0.874; df = 3; *P =* 0.832
Mixed (*n =* 546)	316 (57.9)	196 (35.9)	33 (6.0)	01 (0.2)	
**Source of drinking water [as per participants' response] (*****n** =* **649)**					
Borewell water (*n =* 33)	20 (60.6)	12 (36.4)	01 (3.0)	0	χ^2^= 30.170; df = 9; *P < * 0.001
River water (*n =* 43)	39 (90.7)	04 (9.3)	0	0	
Reverse Osmosis (RO) filtered water (*n =* 139)	87 (62.6)	48 (34.5)	04 (2.9)	0	
Tap water (*n =* 434)	226 (52.1)	171 (39.4)	36 (8.3)	01 (0.02)	
**Height for age (WHO Z score) (*****n** =* **645)**					
Severely stunted (< -3SD) *n =* 56	32 (57.1)	21 (37.5)	03 (5.4)	0	χ^2^= 9.822; df = 9; *P =* 0.365
Stunted (−2SD to −3SD) *n =* 140	78 (55.7)	48 (34.3)	14 (10.0)	0	
Normal (-2SD to +2SD) *n =* 441	259 (58.8)	161 (36.5)	20 (4.5)	01 (0.2)	
Tall (+2SD to +3SD) *n =* 8	02 (25.0)	05 (62.5)	01 (12.5)	0	
**BMI for age (WHO Z scores) (*****n** =* **645)**					
Severe thinness (< -3SD) *n =* 86	43 (50.0)	33 (38.4)	10 (11.6)	0	χ^2^= 14.04; df = 12; *P =* 0.298
Thinness (−2SD to −3SD) *n =* 159	88 (55.4)	63 (39.6)	07 (4.4)	01 (0.06)	
Normal (−2SD to +1SD) *n =* 387	232 (59.9)	134 (34.7)	21 (5.4)	0	
Overweight (+1SD to +2SD) *n =* 7	03 (42.9)	04 (57.1)	0	0	
Obese (+2SD to +3SD) *n =* 6	05 (83.3)	01 (16.7)	0	0	
**Urine fluoride level (*****n** =* **649)**					
< 1 PPM (*n =* 121)	79 (65.3)	39 (32.2)	03 (2.5)	0	χ^2^ = 37.181; df = 12; *P < * 0.001
1 to 2 PPM (*n =* 224)	141 (62.9)	73 (32.6)	10 (4.5)	0	
2 to 3 PPM (*n =* 139)	82 (59.0)	44 (31.6)	13 (9.4)	0	
3 to 4 PPM (*n =* 70)	37 (52.9)	29 (41.4)	04 (5.7)	0	
> 4 PPM (*n =* 95)	33 (34.7)	50 (52.6)	11 (11.6)	1 (1.1)	

As shown in Table 4, with the increasing age of participants, the odds of dental fluorosis were found to increase by 2–4 folds. The odds of having dental fluorosis were significantly increased with increasing water fluoride levels of 3 to 5 PPM [AOR = 3.147 (1.585–6.248); *P* = 0.001] in comparison with water fluoride levels of < 1 PPM. The similar trend was found with urine fluoride level>4 PPM [AOR = 3.607 (1.861–6.990); *P* < 0.001]. Gender, type of diet, height for age and BMI for the age of study participants were not found to be associated (p>0.05) with dental fluorosis. Tap water as a source of drinking water was found most frequently associated with dental fluorosis. Mean and standard deviations of water fluoride levels in samples collected from bore well water, river/pond water, RO filter water and tap water from each village is depicted in Table 5.

**Table 4:** Bivariate and multivariable logistic regression analysis of dental fluorosis with various factors (*n* = 649).

**Variable**	**Bivariate analysis**		**Multivariable analysis**	
	**OR (95 % CI)**	***P*** **-value**	**AOR (95 % CI)**	***P*** **-value**
**Age**		< 0.001	–	< 0.001
**6 years**	1	–	1	–
**7 years**	1.927 (1.053–3.528)	0.033	2.861 (1.469–5.573)	0.002
**8 years**	2.747 (1.477–5.107)	0.001	4.142 (2.087–8.218)	< 0.001
**9 years**	3.461 (1.866–6.421)	< 0.001	6.351 (3.161–12.761)	< 0.001
**10 years**	3.975 (2.194–7.204)	< 0.001	7.570 (3.867–14.821)	< 0.001
**11 years**	3.750 (1.954–7.197)	< 0.001	6.975 (3.368–14.444)	< 0.001
**12 years**	3.624 (1.953– 6.723)	< 0.001	7.282 (3.753–14.840)	< 0.001
**Gender**				
**Male**	1	–	Not included in multivariable model	
**Female**	0.797 (0.583–1.088)	0.153		
**Type of diet**				
**Vegetarian**	1	–	Not included in multivariable model	
**Mixed**	0.867 (0.568–1.324)	0.510		
**Source of drinking water**		< 0.001	–	< 0.001
**River/pond water**	1	–	1	–
**Borewell water**	6.337 (1.827 – 21.978)	0.004	3.622 (0.954 – 13.747)	0.059
**Tap water**	8.973 (3.152 – 25.543)	< 0.001	10.224 (3.494 – 29.921)	< 0.001
**RO water**	5.828 (1.969 – 17.244)	0.001	5.778 (1.879 – 17.767)	0.002
**Height for age (WHO Z scores)** ^**^		0.334		
Severely stunted (< -3SD)	1	–	Not included in multivariable model	
Stunted (−2SD to −3SD)	1.060 (0.567–1.981)	0.856		
Normal (−2SD to +2SD)	0.937 (0.534–1.644)	0.820		
Tall (+2SD to +3SD)	4.00 (0.741–21.582)	0.107		
**BMI for age (WHO Z scores)** ^**^		0.264		
Severe thinness (< -3SD)	1	–	Not included in multivariable model	
Thinness (−2SD to −3SD)	0.807 (4.77–1.365)	0.424		
Normal (−2SD to +1SD)	0.668 (0.418–1.068)	0.092		
Overweight (+1SD to +2SD)	1.333 (0.281–6.316)	0.717		
Obese (+2SD to +3SD)	0.20 (0.022–1.784)	0.149		
**Water fluoride level**		< 0.001	–	0.005
< 1 PPM	1	–	1	–
1 to 3 PPM	1.179 (0.845–1.645)	0.333	1.180 (0.750–1.636)	0.608
3 to 5 PPM	3.564 (1.986–6.396)	< 0.001	3.147 (1.585–6.248)	0.001
**Urine fluoride level**		< 0.001	–	< 0.001
< 1 PPM	1	–	1	–
1 to 2 PPM	1.107 (0.697–1.758)	0.666	0.978 (0.596–1.605)	0.930
2 to 3 PPM	1.307 (0.790–2.165)	0.297	1.135 (0.652–1.975)	0.654
3 to 4 PPM	1.678 (0.920–3.058)	0.091	1.548 (0.804–2.890)	0.191
> 4 PPM	3.534 (2.010–6.213)	< 0.001	3.607 (1.861–6.990)	< 0.001

^**^Five participants' height and BMI were missing.

**Table 5:** Water fluoride levels (in PPM) in villages (*n* = 62 samples).

**Taluk**	**Village**	**Borewell water**	**River/ Pond water**	**Tap water**	**RO water**
Manvi taluk	Torandinni	2.31 ± 0.908 (*n =* 5)	-	-	-
	Ballatagi	0.75 ± 0.255 (*n =* 2)	-	-	0.10 ± 0 (*n =* 1)
	Kallur	2.905 ± 0.120 (*n =* 2)	0.396 ± 0.016 (*n =* 2)	-	-
	Pamankallur	1.47 ± 0.562 (*n =* 3)	0.384 ± 0 (*n =* 1)	-	-
	Sirwar	2.68 ± 0.909 (*n =* 4)	-	1.08 ± 0.010 (*n =* 2)	-
	Kurdi	1.19 ± 0.850 (*n =* 5)	-	-	-
	Byagwat	0.37 ± 0 (*n =* 1)	0.33 ± 0 (*n =* 1)	-	0.08 ± 0 (*n =* 1)
	Hirekotnekal	0.43 ± 0.355 (*n =* 3)	-	0.34 ± 0 (*n =* 1)	-
	Pothnal	-	0.85 ± 0.697 (*n =* 2)	-	-
Devadurga	Gandhal	1.63 ± 0 (*n =* 1)	-	-	0.224 ± 0 (*n =* 1)
	Gabbur	0.432 ± 0.283 (*n =* 3)	-	1.605 ± 0.007 (*n =* 2)	-
	Galag	1.56 ± 0.728 (*n =* 2)	-	-	-
	Koppar	2.62 ± 0 (*n =* 1)	-	-	2.5 ± 0 (*n =* 1)
	Masarkal	0.61 ± 0.256 (*n =* 3)	-	0.59 ± 0.125 (*n =* 3)	0.087 ± 0 (*n =* 1)
	Ramadurga	3.28 ± 1.043 (*n =* 4)	-	-	-
	Hire budur	2.32 ± 0 (*n =* 1)	-	-	3.89 ± 0 (*n =* 1)
	Chinchodi	-	-	0.965 ± 0 (*n =* 1)	0.081 ± 0 (*n =* 1)

## Discussion

The present study estimated the prevalence of dental fluorosis to be 46% among 6 to 12 years of children in villages of two taluks of Raichur district of Karnataka. The prevalence of dental fluorosis was found 41.73% in Mysuru among 10 to 12 years old children (17), 64.9% in 9 to 15 years children of Bagalkot (18), 64.3% in 12 to 17 years children of Kolar (11), and 73% in 3 to 17 years children of Vijaypura (19) districts of Karnataka in earlier studies. The prevalence of dental fluorosis has increased in school children of the Raichur district from 32.6 to 46% in the last decade. The difference in the prevalence of various regions of Karnataka may be attributed more to the wide range of age groups included in these studies and less to the variation in fluoride levels in drinking water. The increasing prevalence in the Raichur district requires immediate attention for improving the dental health of the children.

Amongst all children affected by fluorosis, most of them were having mild (37.9%) and moderate fluorosis (7.8%); only 0.3% of children had severe fluorosis in the present study. The prevalence of severe fluorosis was higher (8.1 to 18.6%) in other studies (9, 18, 20) which may be due to variation between age groups included in the study. More advanced age group children were included in these studies as compared to 6 to 12 years children in the present study.

In the present study, the prevalence of fluorosis was found to increase with the increase in the age of the study participants which may be due to the irreversibility of developed effects of dental fluorosis (21). Fluoride exposure during the first 2 years of life is an important risk factor for the development of fluorosis in permanent central incisors (22) however, late erupting teeth have a higher risk of developing fluorosis up to the age of 8 years (23). Most of the children who are at risk of developing dental fluorosis may develop it by 6–8 years and once developed fluorosis is irreversible. Hence, its prevalence increases with increasing age. During the developmental period, the quantity and duration of fluoride ingestion determine the severity of dental fluorosis (9). Fluoride exposure in children mainly occurs due to drinking water with high fluoride levels, certain foods, and fluoridated toothpaste if ingested frequently by them. Drinking water is the main cause of fluoride exposure. Fluoride is primarily absorbed from drinking water, through the digestive tract and enters the body, and persists as hydrofluoric acid. Also, fluoride may enter the human body through other dietary products as well which have different fluoride ingestion patterns (24). Fluoride accumulates mainly in mineralized tissues like teeth and bones (24). Due to excess fluoride in water dental mottling occurs mostly in permanent teeth and is clearly visible in children above 5 years of age (11).

In the present study, the odds of fluorosis events were found significantly higher with increasing water and urine fluoride levels (Table 4). Moreover, water fluoride levels of >1 ppm were found in 22 water samples from bore wells, 4 samples from tap water, and 2 samples from community RO-filtered water. Excessive fluoride ingestion leads to higher urinary fluoride excretion. The findings of the present study with water and urinary fluoride levels confirm that the exposure to excessive fluoride in the study participants was from drinking water. There was more frequency of dental fluorosis in children whose major source of drinking water was tap water, community filters, and borewell water whereas, it was less frequent with children who had river/pond water as a drinking water source. In this study, fluoride level was found less in river/pond water than in all other listed sources. Moreover, there was a significant association between water fluoride levels and dental fluorosis in the study area that confirms high fluoride levels in the water as a cause of dental fluorosis in our study population. It is further strengthened by the significant association of urine fluoride excretion with dental fluorosis. The type of diet (Veg vs. Non-veg) taken by children did not affect the occurrence of dental fluorosis. Increasing age was associated with increased odds of having dental fluorosis in this study. Similar findings were found in studies conducted by Bhagavatula et al. (23), Dong et al. (25), Shruthi et al. (26), and Saldarriaga et al. (27). In study area, the community filter plants have been installed recently and many households also started using them as the source of drinking water that might have resulted in less fluoride exposure to younger children as compared to older children.

In this study, according to height for age (WHO Z scores), 30.2% of children were stunted, and on BMI for age WHO Z score assessment, 37.8% were underweight and 2.8% were overweight/obese. However, an association of dental fluorosis with height and BMI was found non-significant, whereas Mahantesha et al. (18) found the effect of nutritional status on the severity of fluorosis, with malnourished children being more affected by severe fluorosis. Fluoride exposure can affect the nutritional status and cognitive development of children. In China, low to moderate fluoride exposure was found to be associated with overweight and obesity among 7 to 13 years of children (28). Excess fluoride exposure may negatively affect the BMI (29). The results of a systematic review conducted by Choi et al. (30) supported the possibility of adverse neurodevelopment in children due to excessive fluoride exposure. In the present study, we did not evaluate the intelligence quotient/performance of children.

As the effect of excess fluoride exposure are not limited to dental fluorosis and the effects once developed are irreversible, more focus should be given to preventive strategies. Educating the communities about the ill effects of excessive fluoride in water, empowering them to measure fluoride easily in drinking water, and de-fluoridation of drinking water or diluting the high fluoride water by mixing it with the rainwater are the important steps that may be helpful in prevention. Further, parents of children should be made aware of correct brushing habits with fluorinated toothpaste, which makes sure that children < 6 years of age do not swallow toothpaste while brushing or brushing with non-fluoridated toothpaste. In a study conducted by Gupta et al. (21), administration of ascorbic acid, calcium, and vitamin D3 was found to improve dental, clinical, and skeletal fluorosis in early-grade disease. This treatment strategy may be useful in children during the early stage of fluorosis but, it should be monitored with their serum levels to prevent toxicities of ascorbic acid, calcium, and vitamin D3.

The present study measured the prevalence of clinical dental fluorosis and confirmed it due to fluoride overexposure from drinking water through urine and water fluoride level estimations in an aspirational district of Karnataka. It also focused on association of malnutrition with dental fluorosis.

There were a few limitations of the study. It was conducted as a sub-study of a large study where we could not evaluate the urinary fluoride level amongst all the children in whom fluorosis was clinically evaluated due to inadequate urine samples left for fluoride analysis after the completion of a primary analysis on those samples. Thus, the sample size of the study was therefore compromised. It was a cross-sectional study, so temporal association between duration and extent of fluoride exposure to development of dental fluorosis could not be established.

In conclusion, the prevalence of dental fluorosis was high in 6 to 12 years children in Manvi and Devadurga taluks of Raichur district due to overexposure of fluoride from drinking water. High water and urine fluoride levels in children indicate the chronic exposure to fluoride and suggest that the population is at high risk of developing chronic fluorosis. The measures to provide safe drinking water with permitted fluoride limit should be taken along with creating awareness amongst community about high fluoride level in water and its harmful effects. Community based intervention of ascorbic acid, calcium and vitamin D3 can be planned and evaluated for long term effect of fluorosis.

## Data availability statement

The raw data supporting the conclusions of this article will be made available by the authors, without undue reservation.

## Ethics statement

The studies involving human participants were reviewed and approved by the Institutional Ethics Committee, Raichur Institute of Medical Science, Raichur. Written informed consent to participate in this study was provided by the participants' legal guardian/next of kin.

## Author contributions

UP and PV: project implementation, management, data collection, fluoride analysis, data entry, and first draft of manuscript. PV, CN, and MB: data review, analysis, and drafting and finalizing the manuscript. RK: project implementation, fieldwork management, and finalization of draft. SR: data acquisition through ODK, data management, and finalization of the manuscript. SRa: drafting and finalization of the manuscript. RB and TC: fluoride analysis, revision, and finalization of the manuscript. MM: fieldwork, administrative support, revision, and finalization of the manuscript. SRo: conceptualization, overall project management, field work monitoring, and drafting and finalization of manuscript. All authors contributed to the article and approved the submitted version.
